# Preferred use of contraceptive methods and reasons for non-use: a cross-sectional survey of a sample of Black, Indigenous, and people of colour in the United States

**DOI:** 10.1080/26410397.2025.2494418

**Published:** 2025-04-16

**Authors:** Alexandra Wollum, Katherine Key, Carmela Zuniga, Charon Asetoyer, Maricela Cervantes, Sung Yeon Choimorrow, Raquel Z. Rivera, Janette Robinson Flint, Sarah E. Baum

**Affiliations:** a Research Scientist, Ibis Reproductive Health, Oakland, CA & Cambridge, MA, USA. *Correspondence*: awollum@ibisreproductivehealth.org; allie.wollum@gmail.com; b Associate Research Scientist, Ibis Reproductive Health, Oakland, CA & Cambridge, MA, USA; c Senior Associate Research Scientist, Ibis Reproductive Health, Oakland, CA & Cambridge, MA, USA; d Executive Director/CEO, Native American Community Board, Lake Andes, SD, USA; e Director of Research, California Latinas for Reproductive Justice, Los Angeles, CA, USA; f Executive Director, National Asian Pacific American Women’s Forum, Chicago, IL, USA; g Senior Research and Grants Associate, Bold Futures NM, Albuquerque, NM, USA; h Executive Director, Black Women for Wellness, Los Angeles, CA, USA; i Senior Research Scientist, Ibis Reproductive Health, Oakland, CA & Cambridge, MA, USA

**Keywords:** contraception, preferred contraceptive methods, barriers, person-centred care

## Abstract

Use of preferred contraceptive methods is a measure of reproductive autonomy, yet barriers persist across the United States in accessing preferred methods of contraception, with disparities in access among communities of colour. Using data from a 2021–2022 cross-sectional survey of 727 people aged 13–50 living in the United States who identified as Asian American, Native Hawaiian, or Pacific Islander (29%); Black or African American (34%), Indigenous (13%), and Latina/Latinx (31%), we examined those who were not using their preferred contraceptive method(s), including the preferred method type and the reasons for not using this method(s). We ran an adjusted logistic regression to test the association between the quality of the last health care interaction related to contraception and the use of a non-preferred method. Thirty-seven percent of respondents preferred a contraceptive method they were not currently using. Among current contraceptive users, long-acting methods were preferred most often, while non-current contraceptive users desired long-acting and short-acting hormonal methods equally. Respondents most often cited concerns about side effects/health risks (65%) and financial/logistical reasons (42%) as the top reasons for not using their preferred method(s). Those who reported receiving higher quality care in a recent contraceptive visit were more likely to be using the method they wanted to be using. Use of a preferred contraceptive method may increase when receiving high-quality counselling and care. Strategies to improve access to preferred methods should address side effects and health concerns, as well as financial and logistical barriers among Black, Indigenous, and people of colour.

## Plain language summary

Use of preferred contraceptive methods is a measure of reproductive autonomy. But barriers persist across the United States in accessing contraception. There are disparities in access to contraception among communities of colour. In 2021–2022 we conducted a survey among a convenience sample of 727 people who identified as Asian American, Native Hawaiian, or Pacific Islander, Black or African American, Indigenous, and Latina/Latinx. Thirty-seven percent of respondents would rather use a different contraceptive method than the one currently used.

Most current contraceptive users preferred long-acting methods. People not currently using contraceptives desired long-acting and short-acting hormonal methods equally. Concerns about side effects, health risks, cost and logistics were the main reasons for not using a preferred method. Respondents who recently received higher quality contraceptive care were more likely to be using the method they wanted. High quality contraceptive counselling and care may mean more people use their preferred contraceptive method. Strategies to improve access to preferred methods should address side effects and health concerns, as well as financial and logistical barriers among Black, Indigenous, and people of colour.

## Résumé

L’utilisation des méthodes contraceptives préférées est une mesure de l’autonomie reproductive; pourtant des obstacles persistent dans l’ensemble des États-Unis d’Amérique pour avoir accès aux méthodes de contraception préférées, avec des disparités d’accès entre communautés de couleur. À l’aide des données d’une enquête transversale de 2021–2022 auprès de 727 individus âgés de 13 à 50 ans vivant aux États-Unis qui se sont identifiés comme Américains d’origine asiatique, natifs d’Hawaï ou d’îles du Pacifique (29%), Noirs ou Afro-américains (34%), autochtones (13%), et Latino-Américains (31%), nous avons examiné ceux qui n’utilisaient pas leur(s) méthode(s) préférée(s) de contraception, notamment le type de méthode préférée et les raisons pour lesquelles ils n’utilisaient pas cette méthode. Nous avons effectué une régression logistique ajustée pour tester l’association entre la qualité de la dernière interaction de soins de santé liée à la contraception et l’utilisation d’une méthode non préférée. Trente-sept pour cent des répondants préféraient une méthode contraceptive qu’ils n’utilisaient pas alors. Parmi les utilisateurs de contraceptifs au moment de l’enquête, les méthodes à action prolongée étaient le plus souvent préférées, alors que les utilisateurs de contraceptifs qui n’y avaient pas recours au moment de l’enquête souhaitaient de manière égale des méthodes hormonales à action prolongée et de courte durée. Les personnes interrogées ont cité le plus fréquemment des préoccupations concernant les effets secondaires/risques pour la santé (65%) et des raisons financières/logistiques (42%) comme principaux motifs du non-emploi de leur(s) méthode(s) préférée(s). Celles qui ont déclaré avoir reçu des soins de meilleure qualité au cours d’une récente visite de contraception étaient plus susceptibles d’utiliser la méthode qu’elles souhaitaient. Le recours à une méthode contraceptive préférée peut augmenter en cas de conseils et de soins de qualité. Les stratégies visant à élargir l’accès aux méthodes préférées doivent tenir compte des effets secondaires et des préoccupations de santé, ainsi que des obstacles financiers et logistiques parmi les personnes noires, autochtones et de couleur.

## Resumen

El uso de métodos anticonceptivos preferidos es una medida de autonomía reproductiva; sin embargo, en Estados Unidos (EE. UU.) persisten las barreras al acceso a métodos anticonceptivos preferidos, con disparidades en el acceso entre comunidades de color. Utilizando los datos de una encuesta transversal administrada en 2021–2022 a 727 personas entre 13 y 50 años, que vivían en EE. UU. y se identificaron como asiáticas americanas, indígenas hawaianas o isleñas del Pacífico (29%); negras o afroamericanas (34%), indígenas (13%) y latinas (31%), examinamos a las personas que no estaban usando su(s) método(s) anticonceptivo(s) preferido(s), así como el tipo de método preferido y las razones para no usar el(los) método(s). Realizamos una regresión logística ajustada para probar la asociación entre la calidad de la última interacción con servicios de salud relacionada con la anticoncepción y el uso de un método no preferido. El 37% de las personas encuestadas prefirieron un método anticonceptivo que no estaban usando actualmente. Las usuarias de anticonceptivos actuales prefirieron con mayor frecuencia los métodos de acción prolongada, mientras que las usuarias de anticonceptivos no actuales deseaban por igual métodos hormonales de acción prolongada y de corta duración. Como las principales razones para no usar su(s) método(s) preferido(s), las personas citaron con mayor frecuencia preocupaciones sobre los efectos secundarios/riesgos para la salud (65%) y razones financieras/logísticas (42%). Aquéllas que indicaron haber recibido atención de mayor calidad en una consulta anticonceptiva reciente eran más propensas a estar usando el método que querían usar. El uso de un método anticonceptivo preferido podría aumentar cuando se recibe consejería y atención de alta calidad. Las estrategias para mejorar el acceso a métodos preferidos deberían abordar los efectos secundarios y las preocupaciones de salud, así como las barreras financieras y logísticas entre las personas negras, indígenas y de color.

## Introduction

Use of a preferred contraceptive method is a key measure of reproductive autonomy and choice, yet among the 65% of pregnancy-capable people using a contraceptive method in the United States (US), a quarter report not using their preferred method.^1,2^ Multiple factors influence contraceptive method preferences, such as knowledge and perceptions of methods, previous experiences, method effectiveness, contraceptive counselling, concerns about side effects and overall health impact, ease of use, and partner preference.^3^ Access to contraceptive methods, and barriers such as a lack of availability of methods, cost, lack of insurance coverage, prescription requirements, and difficulty reaching a provider, can also play a role in use of preferred methods. These barriers disproportionately impact certain groups of people; disparities in use of a preferred method exist based on age, ethnicity, and income, with young people (18–25 years), Hispanic individuals, and those with lower incomes more likely to report not using their preferred method, compared to their counterparts.^2,4,5^

Method preferences among Black, Indigenous, and people of colour may also be influenced and shaped in part by historical injustices and ongoing disparities in access and quality of reproductive health care, including historical unethical experimental studies which tested oral contraceptive pills on Puerto Ricans and Depo Provera on Native women, the study of obstetrics and gynaecology using enslaved Black women’s bodies,^6^ persisting reproductive coercion and low quality reproductive health care, forced sterilisation of communities of colour, and the ongoing dismissal of Black women’s pain.^7–12^ Previous literature has documented that contraceptive method feature preferences vary by racial and ethnic identity, with Black and Latina/x women identifying features such as being able to stop the method at any time and having control over when and whether to use the method as more important than White women.^13^ In addition, access to contraceptive care may be influenced by structural barriers including systematic racism and oppression, policies that limit access to publicly funded contraceptive care, and limited public transportation and affordable childcare.^14–16^ These barriers disproportionately impact Black, Indigenous, and people of colour and resulting disparities in insurance coverage, degree of autonomy in seeking contraceptive care, and availability of contraceptive methods may prevent many from obtaining their preferred contraceptive method.^8,17^ Given the intersecting factors which influence contraceptive method preferences and barriers to contraceptive access for Black, Indigenous, and communities of colour, it is important to centre their voices to better understand alignment between contraceptive use and preferences.

The majority of research on use of non-preferred methods focuses on cost and lack of insurance as a major barrier and reason for non-use,^4,18^ but barriers may be broader and include method (dis)satisfaction,^19^ concern about side effects,^2,4^ and poor quality interactions with medical providers.^2,20^ A deeper examination of how interactions with providers offering contraceptive care influence use of a preferred method is particularly important for Black, Indigenous, and people of colour given the large body of literature pointing to non-person centred and coercive care that often pushes people toward specific methods (often long-acting or permanent methods).^21–26^ For individuals not currently using contraception but who would like to be, it is unknown if the barriers they encounter to obtaining a preferred contraceptive method are different, more numerous, or more difficult to overcome than those who are currently using a non-preferred method. This population is included in only limited literature exploring use of a preferred contraceptive method.^2,27,28^ Moreover, the past literature on use of non-preferred contraceptive methods has not explored reasons for non-use by the type of contraceptive method preferred. Understanding how barriers to preferred method use vary by preferred method is critical to design targeted interventions, programmes, and policies that help people align their preferences with use.

This study aimed to document use of preferred contraceptive methods, the reasons for not using preferred methods, and the relationship between the quality of health care interactions and preferred method use, specifically among Asian American, Native Hawaiian, and/or Pacific Islander; Black or African American; Latina/Latinx; and Indigenous individuals in the US.

## Methods

### Study design

From May 2021 to March 2022, we conducted a cross-sectional survey with people who identified with at least one of the following racial or ethnic groups: Asian American, Native Hawaiian, or Pacific Islander (AANHPI), Black or African American, Indigenous, and Latina/Latinx. This study utilised a community-engaged approach in which a collaborative research team, consisting of six organisations with research and/or advocacy expertise, designed and implemented the study. Of the six organisations, five work directly with and include leadership and representation from communities included in this study. AW is a public health social science researcher studying preferences for, access to, and quality of abortion and contraceptive care who identifies as white and is employed at a US-based research non-profit. KK is a social science researcher who identifies as Asian and White and works at a non-profit conducting quantitative research focused on expanding access to contraception and safe abortion care in the US and globally. CZ is a social science researcher who identifies as Asian and Latina and who works at a nonprofit focused on advancing sexual and reproductive health. CH is a member of the Comanche Nation and a reproductive justice activist that has 35 years of experience working to improve the health and wellbeing of Indigenous women. MC is a public health practitioner and researcher who identifies as a queer Latina raised by a family of immigrants in American poverty. MC is the Associate Director at a US-based Reproductive Justice Organization. SYC is the executive director of a national nonprofit that builds collective power with Asian American and Pacific Islander women and girls. She identifies as a first-generation, Korean American, immigrant working mom. RZR is a policy researcher at a reproductive justice non-profit based in New Mexico, USA. Born and raised in Puerto Rico, New Mexico has been home since she birthed her only child at 40 years old with Medicaid coverage and assisted by midwives at her family residence in Albuquerque’s South Valley. JRF is an African American health educator working as the Executive Director of Black Women for Wellness, a community-based organisation centring Black women and girls with research, programme and policy to eliminate health inequities impeding our ability to live extraordinary lives with health and abundance. SEB is a white, Jewish, social science researcher who works at a non-profit and focuses on qualitative studies addressing people’s experiences accessing contraception and abortion in the US and globally.

Using convenience sampling, eleven community-based organisations and two data collectors based in California, Georgia, Illinois, and New Mexico recruited respondents using online invitations and in-person methods. Due to the location of organisations involved in recruitment, nearly 60% of respondents were located in these states. The survey was available in four languages – English, Spanish, Vietnamese, and Urdu – chosen by the collaborative research team to ensure and increase participation among communities they serve. We aimed to recruit 200 people in each racial or ethnic group to enable analysing data within racial and ethnic groups.

People were eligible to participate if they lived in the US; were between the ages of 13 and 50; identified as AANHPI, Black or African American, Indigenous, and/or Latina/Latinx; identified as a woman, transgender, or non-binary, regardless of sex assigned at birth; had used or wanted to use any contraceptive method (hormonal or non-hormonal) in the past year; and had a functioning email address to receive a link to the survey. Each participant who completed the survey received a $25 gift card; however, due to a high volume of fraudulent activity and online bots, we developed a rigorous, multi-step review process to determine the eligibility of each respondent to receive the gift card and be included in our final sample.^29^

The study was approved by the Allendale Investigational Review Board [Protocol #: CUP032021, on March 11, 2021]. Participants provided informed consent through a question in the Qualtrics survey after viewing information about the survey. Participants under 18 were not required to obtain parental consent. Participants were given study staff contact information if they wanted to ask questions about the study. Additional findings from this study have been published previously.^29^

### Variables

We used the question asked of all respondents “Is there a method of birth control that you would like to use but you are not currently using?” to understand the alignment between contraceptive use and preferences. Respondents who answered “yes” were considered not to be using their preferred method and were asked which method(s) they wanted to be using, the reasons they were not currently using the method(s) they wanted to be using from a set of options (See Appendix Table A1), and an open-ended question on what would help to use the method(s) they wanted to use. We categorised the methods preferred into six categories: long-acting reversible contraceptive methods (LARCs) (i.e. implants, copper IUDs, and hormonal IUDs), short acting reversible contraceptive methods (SARCs) (i.e. oral contraceptives, ring, patch, injectables), permanent methods (e.g. tubal ligation and vasectomy), barrier methods (i.e. external condoms, internal condoms, vaginal barrier methods), natural methods (i.e. withdrawal, fertility awareness methods), and emergency contraception. We included respondents in multiple categories where they reported preferring more than one method.

We created six categories describing the reasons selected in the close-ended question about why participants were not using their preferred method(s): method-related reasons; logistical/financial related reasons; sexual relationship related reasons; situational/relational reasons; provider-related reasons; and pregnancy/birth related reasons. (See Appendix Table A1).

Among respondents who reported visiting a provider or pharmacist about contraception in the past year, we constructed an index capturing the quality of the last health care interaction with a provider (clinician or pharmacist) about birth control. The index captured the quality of the interpersonal interaction with the provider and was constructed by taking the average across four measures (See Appendix Table A2 and Appendix Figure A1). Higher values represented higher quality care and the index ranged from 1 to 5. Three of the four measures were drawn from the interpersonal quality of family planning care scale and the fourth was drawn from the Reproductive Health Impact Study.^30,31^

To characterise demographics, we examined respondents’ self-identified race and ethnicity, age, educational attainment, insurance status and type, whether they received services from Indian Health Service (IHS), whether they were born in the US, whether they had given birth, their sexual orientation, and their gender identity. We used the zip code provided by respondents in the survey to identify their region of residence within the US using the census Regions.^32^ We categorised respondents who said they had used any method of birth control in the past month as current contraceptive users. We grouped contraceptive methods that respondents reported using in the past month in the same categories reported above (LARCs, SARCs, permanent methods, barrier methods, natural methods, and emergency contraception). We included respondents in multiple categories where they reported using more than one method.

### Analysis

We calculated the proportion of people who wanted to be using a different method of birth control among the overall sample and among current contraceptive users and non-current contraceptive users by respondent characteristics. We then summarised the proportion of respondents who wanted to be using a different method(s) within contraceptive type categories, examining preferred method types by contraceptive method type used in the past month. We then summarised the percentage of respondents who selected each overarching reason category for not using their preferred method, as well as the individual reasons within categories. We examined reasons among the full sample, by method type preferred, and by current and non-current contraceptive users. We included missing data in our analyses for categorical variables by creating specific categories where the percentage of missing data exceeded 3%. For variables that were continuous, we excluded missing data from our analyses. Less than 1% of the sample was missing data on whether they were using their preferred method.

We coded open-ended responses to the question, “What would help you use the method(s) you want to use?”.^33,34^ We created approximately 12 categories based on review of responses and *a priori* topic areas related to overcoming barriers such as side effects, interpersonal interactions, and logistics. For example, categories included information about contraceptive methods (risks, side effects), access to a provider, financial support/insurance coverage. We coded each open response based on these categories; one response could be assigned to more than one category. Two authors reviewed all responses.

Finally, we constructed a multivariable model to understand how the quality of the last health care interaction was associated with non-preferred method use. We included the interpersonal quality index as a continuous predictor in a logistic regression model and controlled for age, race and ethnicity, insurance coverage, gender identity, and nativity. Covariates were selected based on their hypothesised relationship between quality of care and non-preferred method use. We tested for model goodness of fit using a Hosmer-Lemeshow test. We ran the model among the full sample of respondents who reported they had interacted with a clinician or pharmacist in the past year about birth control and among current contraceptive users and non-current contraceptive users separately. We do not report on the models with non-current contraceptive users, given the small sample size.

Analysis was conducted in Stata 15 SE.

## Results

### Characteristics of sample

A total of 727 respondents were included in our final sample (Table 1). Nearly a third of respondents identified as each of the following three identities: Hispanic or Latina/Latinx, AANHPI, or Black or African American. An additional 13% identified as American Indian or Alaskan Native. Nine percent of respondents also identified as White and 14% of respondents selected more than one race or ethnicity. On average, respondents were 26.5 years old with ages ranging from 14 to 50. Nearly half (47%) of respondents had college degrees and 16% had professional or advanced degrees. A fifth of the sample (20%) was born outside of the US and 15.1% reported having public health insurance. Seventeen percent reported being uninsured. A quarter of the sample reported not using contraception in the month prior to the survey (25%) while similar proportions reported using LARCs (24%) and SARCs (30%).

**Table 1.** Characteristics of the sample of respondents

	Sample characteristics	
	*Full sample*	
	*n*	%
	727	100
Race/ethnicity*		
*American Indian or Alaskan Native*		
Yes	92	12.7%
No	635	87.3%
Missing	0	0.0%
*Asian American, Native Hawaiian, and/or Pacific Islander*		
Yes	213	29.3%
No	514	70.7%
Missing	0	0.0%
*Black or African American*		
Yes	245	33.7%
No	482	66.3%
Missing	0	0.0%
*Hispanic, Latina, Latinx, or Spanish origin*		
Yes	228	31.4%
No	499	68.6%
Missing	0	0.0%
*White†*		
Yes	63	8.7%
No	664	91.3%
Missing	0	0.0%
**Age, years**		
Under 25	339	46.6%
25–34	300	41.3%
35+	88	12.1%
Missing	0	0.0%
**Education level completed**		
Less than college	266	36.6%
College graduate	340	46.8%
Professional or advanced degree	118	16.2%
Missing	3	0.4%
**Insurance type**		
Public (Medicaid/Medical)	110	15.1%
Private	473	65.1%
None	121	16.6%
Don’t know/Missing	23	3.2%
**Receives services from Indian Health Service**		
Yes	50	6.9%
No	654	90.0%
Unknown	23	3.2%
**Born in US**		
Yes	580	79.8%
No	144	19.8%
Missing	3	0.4%
**Ever given birth**		
Yes	160	22.0%
No	566	77.9%
Missing	1	0.1%
Sexual orientation*		
Straight/heterosexual	486	66.9%
Bisexual	151	20.8%
Gay/Lesbian	11	1.5%
Pansexual	46	6.3%
Queer	85	11.7%
Asexual	23	3.2%
Questioning	39	5.4%
Same gender loving	9	1.2%
Other	8	1.1%
Missing	7	1.0%
**Gender identity**		
Ciswoman or woman	685	94.2%
Gender expansive	19	2.6%
Multiple	23	3.2%
Missing	0	0.0%
**Region of country**		
Northeast	50	6.9%
Midwest	120	16.5%
South	253	34.8%
West	303	41.7%
Missing	1	0.1%

Note: * Select all that apply

† Respondents who selected White as one of their racial identities also selected another racial or ethnic identity due to eligibility criteria for the study.

### Prevalence of non-use of preferred method

Thirty-seven percent of respondents reported there was at least one method they wanted to use that they were not currently using (Table 2). This proportion was higher among respondents who were not using a method of contraception in the month prior to the survey (53% compared to 32% among current contraceptive users). Among current contraception users and non-current users, respondents under 25 were more likely not to be using their preferred method than older participants (44% vs 31% and 26% among 25–34 and 35+ participants respectively) and those who did not identify as straight or heterosexual more often reported not using their preferred method than those who identified this way (43% vs 34%). Respondents who identified as Black or African American were more likely to be using their preferred contraceptive method compared to respondents who did not identify as Black or African American (30% vs 40%) (Table 2). Participants who identified as cis-gender more often reported using their preferred contraceptive method compared to respondents who identified as gender-expansive (53% vs 36% were not using their preferred method); however, the sample size was small. We did not find differences by other participant characteristics.

**Table 2.** Percentage of respondents who would like to use a different method of contraception they are not currently using

	All respondents		Current contraceptive users		Non-current users	
	*n*	%	*N*	%	*N*	%
All respondents	266	36.6%	174	31.8%	92	52.9%
Race/ethnicity*						
*American Indian or Alaskan Native*						
Yes	35	38.0%	24	35.3%	11	45.8%
No	231	36.4%	150	31.3%	81	54.0%
*Asian American, Native Hawaiian, and/or Pacific Islander*						
Yes	88	41.3%	56	36.1%	32	60.4%
No	178	34.6%	118	30.1%	60	49.6%
*Black or African American*						
Yes	74	30.2%	48	26.1%	26	43.3%
No	192	39.8%	126	34.7%	66	57.9%
*Hispanic, Latina, Latinx, or Spanish origin*						
Yes	90	39.5%	59	32.4%	31	67.4%
No	176	35.3%	115	31.5%	61	47.7%
*White†*						
Yes	27	42.9%	19	38.8%	8	57.1%
No	239	36.0%	155	31.1%	84	52.5%
**Age, years**						
Under 25	150	44.2%	88	37.0%	62	63.3%
25–34	93	31.0%	71	29.0%	22	42.3%
35+	23	26.1%	15	23.4%	8	33.3%
**Education level completed**						
Less than college	106	39.8%	60	34.1%	46	53.5%
College graduate	119	35.0%	84	31.2%	35	50.7%
Professional or advanced degree	40	33.9%	30	29.7%	10	58.8%
**Insurance type**						
Public (Medicaid/Medical)	39	35.5%	25	31.3%	14	48.3%
Private	172	36.4%	117	32.0%	55	52.4%
None	43	35.5%	29	31.5%	14	53.8%
Don’t know/Missing	12	52.2%	3	33.3%	9	64.3%
**Receives services from Indian Health Service**						
Yes	18	36.0%	14	40.0%	4	26.7%
No	236	36.1%	157	31.2%	79	54.5%
Unknown	12	52.2%	3	33.3%	9	64.3%
**Born in US**						
Yes	204	35.2%	437	30.2%	143	50.4%
No	61	42.4%	99	34.3%	45	60.0%
Missing	1	33.3%	3	33.3%	0	–
**Ever given birth**						
Yes	43	26.9%	32	26.4%	11	28.2%
No	222	39.2%	141	33.2%	81	60.0%
Sexual orientation*						
Straight/heterosexual	163	33.5%	103	28.4%	60	51.3%
Bisexual	65	43.0%	44	36.7%	21	67.7%
Gay/Lesbian	1	9.1%	0	0.0%	1	50.0%
Pansexual	19	41.3%	12	33.3%	7	70.0%
Queer	37	43.5%	23	35.4%	14	70.0%
Asexual	13	56.5%	10	55.6%	3	60.0%
Questioning	19	48.7%	12	42.9%	7	63.6%
Same gender loving	1	11.1%	1	12.5%	0	0.0%
Other	2	25.0%	1	16.7%	1	50.0%
**Gender identity**						
Ciswoman or woman	247	36.1%	162	31.5%	85	51.5%
Gender expansive	10	52.6%	4	33.3%	6	85.7%
Multiple	9	39.1%	8	38.1%	1	50.0%
**Region of country**						
Northeast	20	40.0%	18	45.0%	2	20.0%
Midwest	43	35.8%	24	28.9%	19	52.8%
South	94	37.2%	57	30.8%	37	56.9%
West	108	35.6%	75	31.3%	33	53.2%

Note: Percentages represent row percentages.

† Respondents who selected White as one of their racial identities also selected another racial or ethnic identity due to eligibility criteria for the study.

### Type of method preferred among current users

**Figure 1.**
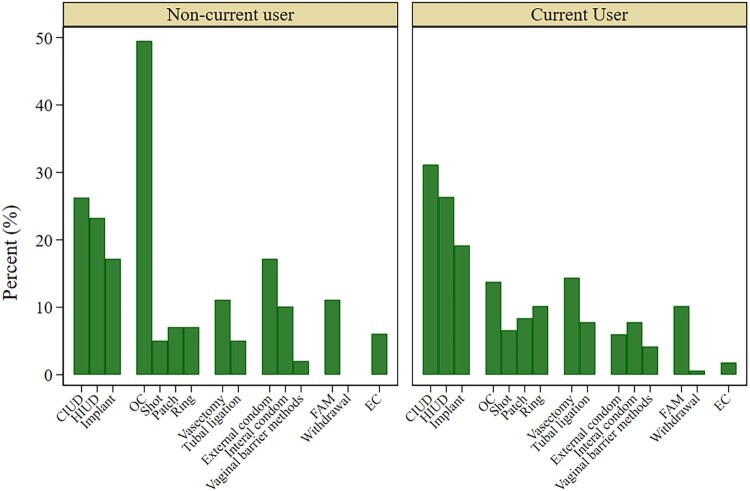
Preferred method among people not using their preferred method, by current method use (*N* = 266). Height of bars represent the proportion of people who preferred to be using the given method among those who were not using their preferred method(s). CIUD: Copper IUD; HIUD: Hormonal IUD; OC: Oral Contraception; FAM: Fertility Awareness Methods; EC: Emergency contraception

Among current contraceptive users who were not using their preferred method, the largest proportion of respondents wanted to be using a LARC (56%) with most respondents preferring a copper IUD (31%) (Figure 1). Twenty nine percent of respondents wanted to use a SARC, with oral contraceptives being the most popular method in this category (14%). Twenty two percent preferred to use a permanent method with vasectomy preferred over tubal ligations and 16% preferred barrier methods. Forty-three percent of respondents who wanted to be using a different method selected more than one method they wanted to be using. The majority of these respondents preferred two (56%) or three methods (27%) and the majority selected methods in multiple method categories (73%).

Considering method preferences by current method use, the largest proportion of current contraceptive users wanted to be using LARC methods, with the exception of barrier method users, who had more equal interest in LARC methods and SARC methods (Table 3). LARC users had the lowest proportion of respondents who preferred to use another method (23%) while respondents using barrier methods or EC in the past month most often reported wanting to use a method they were not currently using (44% and 54% respectively). Importantly, a sizeable proportion of users preferred to switch to other methods in the same category – 12% of LARC users wanted to be using a different LARC than they were currently using.

**Table 3.** Preferred method type by current method used

			Current method type						
			LARC	SARC	Barrier	Natural	Permanent	EC	Not using contraception
Preferred method(s)									
	**Does not prefer a(another) method**		77.5%	67.4%	56.5%	59.7%	100.0%	45.8%	47.1%
	**Prefers a(another) method**		22.5%	32.3%	43.5%	40.3%	0.0%	54.2%	52.9%
		LARC	12.4%	19.7%	24.4%	21.7%	0.0%	33.3%	27.0%
		SARC	7.3%	7.8%	18.5%	11.6%	0.0%	16.7%	31.0%
		Barrier	2.8%	4.1%	6.5%	10.9%	0.0%	8.3%	12.6%
		Natural	2.8%	2.3%	2.4%	4.7%	0.0%	4.2%	6.3%
		Permanent	7.3%	5.5%	8.9%	6.2%	0.0%	0.0%	7.5%
		EC	1.1%	0.5%	0.6%	0.0%	0.0%	0.0%	3.5%

Note: Table displays column percentages.

Respondents could select more than one preferred method such that the per cent of people that prefer each method type does not necessary add up to the total that prefer a method that they are not currently using. Respondents who were currently using a method in the same category of their preferred method are those that prefer to be using a different method in the same method category (e.g. using a hormonal IUD but prefer an implant).

### Type of method preferred among non-current users

Among non-current contraceptive users who wanted to be using a different method, a similar proportion wanted to be using a LARC method (51%) and short acting methods (59%) with the most popular methods selected being copper (26%) and hormonal IUDs (23%) and oral contraceptives (49%) (Figure 1). Twenty four percent of non-current users wanted to be using barrier methods. Fifty-two percent of non-current users with unfulfilled method preferences selected wanting more than one method. Among the respondents who preferred more than one method, 85% named methods across different method categories.

### Reasons for not using a preferred method

Respondents most often cited concerns about side effects or health risks as the primary reason for not using their preferred method(s) (64%) (Table 4). This was most commonly a barrier for LARCs and SARCs and less commonly a concern for permanent, barrier, or natural methods. However, respondents who selected method-related concerns also selected other reasons; only 18% of the sample selected *only* method-related reasons. The second most common reason was logistical/financial barriers including that the method was too expensive or that the respondent lacked insurance (42%). This was most prevalent for people preferring permanent methods of contraception (64%) but was also cited by nearly 50% of those who wanted to be using a LARC or SARC method. Thirty two percent of respondents selected reasons related to their sexual relationships as reasons for not using their preferred method; most commonly this was infrequent sex, which was particularly high among those preferring barrier methods (49%). While partner opposition was only named by 6% of respondents, 20% of those who wanted to use barrier methods noted this reason. Situational and relational concerns were selected by 28% of respondents which most commonly included the fear of being judged and not feeling comfortable or safe getting the method. Provider-related reasons were named by 12% of respondents, most commonly among permanent method users (20%) with 16% reporting their provider advised them against it. Sixty percent of all respondents not using their preferred method(s) selected more than one reason and the vast majority of these fell into different reason categories (93%). Compared to current users, non-current users were more likely to report infrequent sex (51% vs 16%), challenges with transportation, not having a regular doctor, and experiencing unfair treatment at a doctor’s office as reasons for non-use of preferred method(s). Current users more often noted that their provider advised them against using the method (12% versus 4%). (See Appendix Tables A3 and A4).

**Table 4.** Reasons for not using preferred method(s) among people not using preferred method, by type of preferred method

	Preferred method type					
Reasons	Overall (*N* = 266)	LARC (*N* = 144)	SARC (*N* = 110)	Permanent (*N* = 50)	Barrier (*N* = 51)	Natural (*N* = 28)
**Method-related**	65.0%	81.3%	72.7%	58.0%	43.1%	35.7%
Worried about side effects and/or health risks	63.9%	80.6%	71.8%	58.0%	43.1%	32.1%
Need more information about methods	3.0%	2.1%	1.8%	4.0%	0.0%	10.7%
**Logistical/financial**	42.1%	45.8%	48.2%	64.0%	33.3%	28.6%
Too expensive and/or no insurance	25.2%	25.0%	29.1%	44.0%	23.5%	28.6%
Challenges with transportation	9.0%	11.1%	12.7%	8.0%	3.9%	7.1%
Challenge making an appointment	15.0%	17.4%	16.4%	18.0%	11.8%	3.6%
Don’t have a regular doctor or clinic	11.3%	14.6%	15.5%	10.0%	11.8%	14.3%
**Sexual relationships**	31.6%	27.8%	33.6%	36.0%	60.8%	39.3%
Partner won’t let me or doesn’t want me to	6.0%	1.4%	4.5%	8.0%	19.6%	7.1%
Infrequent sex	27.8%	27.1%	31.8%	32.0%	49.0%	32.1%
**Situational/relational**	28.2%	26.4%	36.4%	22.0%	29.4%	25.0%
Privacy concerns	10.5%	8.3%	16.4%	8.0%	13.7%	7.1%
Fear being judged	14.3%	10.4%	20.0%	12.0%	19.6%	21.4%
Didn’t feel comfortable/safe getting my method	15.4%	18.1%	16.4%	12.0%	13.7%	7.1%
**Provider-related**	12.0%	13.2%	10.0%	20.0%	13.7%	14.3%
My provider advised me against it	9.4%	10.4%	7.3%	16.0%	7.8%	10.7%
Treated unfairly by staff at the doctor’s office	3.4%	3.5%	4.5%	4.0%	7.8%	3.6%
Couldn’t use my preferred language when talking	0.4%	0.0%	0.9%	0.0%	0.0%	0.0%
**Trying to get pregnant/currently pregnant/just had baby**	**1**.**9%**	**1**.**4%**	**0**.**9%**	**4**.**0%**	**3**.**9%**	**7**.**1%**
**Other**						
Covid-19-related reason	3.4%	4.2%	1.8%	4.0%	2.0%	0.0%
Other	3.4%	2.8%	3.6%	4.0%	2.0%	14.3%
Prefer not to answer	1.5%	1.4%	1.8%	2.0%	0.0%	3.6%

Note: Respondents could select all specific reasons that applied.

We did not examine those who preferred emergency contraception separately given a small sample size (*n* = 9).

When respondents were asked in an open-ended question what would help them use their preferred method, 40% of those who preferred a(another) method they were not using said they wanted more information about contraceptive methods, especially risks and side effects of different methods. Twenty percent of respondents said they needed access to a provider, 13% needed financial support or insurance coverage, and 10% wanted reassurance or someone to help them manage their fears and concerns related to LARC insertion and removal. Nearly 8% of respondents said seeing a provider that would not pass judgment or deny them specific methods was necessary for them to access the method they wanted, and a similar proportion mentioned wanting methods with fewer side effects or impacts on their health and wanting to have more control over who was involved in their decision making (e.g. parents, partners).

### Association between the quality of contraceptive care and not using a desired method

Sixty-two percent of respondents had seen a provider – clinician or pharmacist – in the past year for any contraceptive-related visit. Across the questions included in the interpersonal quality index, respondents rated their care most highly when asked if the provider kept their information private (98% said the care they received was generally good), while respondents were less satisfied with the provider asking about what mattered to them about their birth control method and whether the provider provided enough information to make the best decision (Appendix Table A2). Taking the latter measure, 31% said this aspect of their care was generally negative. Across participants, the index ranged from 1.25 to 5 with an average of 3.8 (Cronbach’s alpha = .82).

In unadjusted and adjusted analyses, we found those who reported receiving higher quality care were more likely to be using their preferred method(s). Specifically, we found that every one unit increase in the interpersonal quality index was associated with a 30% decrease in the odds of preferring to use another method (95% CI: 13%–44%, *p* = .002) (Table 5). This translates to a difference of 32 percentage points in the per cent of people wanting to use another method between those who rate their care as “poor” on all questions in the index and those who rate their care as “excellent” across all the measures. When looking only among those currently using a contraceptive method, results were similar (Table 5).

**Table 5.** Model results testing association between quality of interaction with provider and wanting to use a different method of birth control than currently using

	All users (*N* = 399) OR (95% CI, *p*-value)		Current contraceptive users (*N* = 316) OR (95% CI, *p*-value)	
	Unadjusted	Adjusted	Unadjusted	Adjusted
Interpersonal quality index	0.70** (.56–.86, *p* = .001)	0.70*** (.56–.87, *p* < .001)	0.71** (.55–.91, *p* = .006)	0.71** (.55–.92, *p* = .009)

* *p* < .05 ** *p* < .01 *** *p* < .001

Note: Adjusted models controlled for age, insurance type, race and ethnicity, born in the US, and gender identity. + Race and ethnicity variables were not mutually exclusive categories and respondents could be included in more than one group.

## Discussion

We explore the alignment between method preferences and use among a sample of people who identify as AANHPI, Black or African American, Indigenous, and Latina/Latinx. This study is among only a few studies to include non-current contraceptive users in exploring preferred method use and the first study to explore reasons for non-use of a preferred method by preferred contraceptive method type.

Although this was a convenience sample, and results cannot be generalised to any broader population, we found similarities and differences in our results versus other studies that did use systematic sampling techniques. We found that over a third of respondents were not using their preferred method(s). Compared to most previous studies, we found an elevated proportion of current contraceptive users wanted to be using a different contraceptive method than what they were currently using. In nationally representative studies, a quarter of current contraceptive users were not using their preferred method^2,4^ compared to nearly a third of current contraceptive users in this study. This may be because we had a higher number of young people in our sample who in other studies have been more likely to not have their contraceptive preferences met,^4,28^ or due to barriers to care during the COVID-19 pandemic, or differences in how the question was asked, or it may reflect barriers faced by the AANHPI, Black or African American, Indigenous, and Latina/Latinx communities included in this study. Our results were similar to a nationally representative study that found 36% of women had a contraceptive use-preference mismatch^27^; however, an elevated proportion of non-current contraceptive users in our study reported a contraceptive use-preference mismatch (52% vs 35%).

We found that among our sample of Black, Indigenous, and people of colour who wanted to be using a(another) method, the largest proportion of respondents wanted to be using a(another) LARC method, similar to other studies on this topic.^20,28^ However, many respondents were concerned about the potential side effects or health risks associated with LARC methods. Our analysis shows that providing more information about the potential side effects, including pain and potential ways to manage or eliminate it during insertion and removals of LARCs is an important step to enable people to use the method they prefer. In parallel, people should be offered pain management options such as expanding the use of local anaesthesia and sedation options.^35^ Prior research on IUDs has highlighted significant fear of pain during LARC insertion as a barrier to use.^36^ Attention to this issue among communities of colour is essential given higher anticipated pain at IUD insertion among Black adolescents^37^ and the dismissal of pain experienced by Black communities by the medical community.^11^ And, while LARCs were the most preferred method type, any attempt to increase information and access to LARCs must ensure that LARCs are not preferentially favoured over other methods as they have been historically, particularly for communities of colour, and that people can access and afford removal services when desired.^38^ This is particularly important given that in our sample of non-current contraceptive users, preferences for short-acting hormonal methods outpaced that of LARCs.

Having a recent high-quality interaction with a clinician or pharmacist about birth control was associated with the use of a preferred method. This suggests the importance of centring high quality person-centred care for people that desire an interaction with a provider that prioritises individual values and preferences in counselling and provision of comprehensive information, in order for people to realise their contraceptive desires. There is a large body of literature documenting coercive and non-person-centred contraceptive care for Black, Indigenous, and people of colour.^22–24,39,40^ Our findings demonstrate quantitatively how treatment from a provider can influence the realisation of contraceptive desires and contraceptive autonomy,^41^ a key component of reproductive autonomy, adding to recent literature among nationally representative populations.^2,20,26^ Interventions are needed to ensure that clinicians provide non-judgmental, person-centred care that sufficiently explores the questions that people have about methods and side effects and respects the autonomy of the patient, as well as addresses the other considerations and concerns underlying reasons for non-use of a preferred contraceptive method. These interventions must counteract ways in which structural factors have historically influenced the care that Black, Indigenous, and people of colour receive. Focusing on racial and ethnic concordance with contraceptive providers may also be an important strategy to pursue further.^42^

This study highlights the complexity of the reasons underlying non-use of a preferred contraceptive method. The most common reason listed was around fear of side effects and health concerns related to using a(another) method and this reason was overwhelmingly higher in this study than in previous studies (64% vs. 25% and 29%).^2,4^ Indeed, nationally representative research has found that 50% of contraceptive users would have wanted more information on side effects from their provider before choosing their method and about half of users find side effects more severe than expected (50%), suggesting a gap in information on potential contraceptive side effects, while also acknowledging that fear of side effects may go beyond wanting more information.^4^ Historical injustices enacted on communities of colour alongside poor quality of care^42^ received from providers and lack of comprehensive sex education^43^ may influence how many people feel about the potential health effects of contraception, engendering mistrust and perpetuating fears about specific contraceptive methods. For Native communities in particular, Indian Health Service facilities may not offer a full range of contraceptive options which may limit the information about and access to preferred methods.^44,45^ Fear of side effects and health concerns, however, was rarely listed as the sole reason for not using the preferred method in our sample and many also selected logistical and financial barriers to care, suggesting a continuing need for focus on these barriers to expand access to contraceptive care. Further, the diversity in reasons underlying the mismatch between method use and preferences highlights the need for different strategies depending on method preferences to ensure people can meet their contraceptive desires. For instance, a higher proportion of people wanting to use permanent methods highlighted that their provider advised them against it and that they faced financial and logistical challenges such as the cost and lack of insurance coverage – mirroring challenges highlighted in previous research about denials of sterilisations requests due to provider refusal and insurance^46^ – and that fewer people wanting permanent methods noted concerns about side effects. For people not currently using a contraceptive method, infrequent sex in combination with other barriers and reasons for non-use including fear of side effects may intersect to prevent preferred method use.

This study has several limitations. First, the study is not a representative sample and therefore cannot be generalised to any broader population. We could not analyse the reason or primary reason for non-use of preferred method by type of method(s) as the respondents were not asked to specify reasons by preferred method or specify if one barrier was more salient. In our analysis of quality of care during the last health care interaction, we do not know how use of the method reported in the past month (or lack of use) aligns with the interaction with the health care providers. It may be that someone was using the reported method prior to the visit or was prescribed the method during the visit. We also do not know the type of provider a client visited during this interaction (e.g. a pharmacist versus clinic-based clinician or their demographic characteristics) which may affect the types of contraception they had available and their interaction. Importantly, contraceptive preferences may be based on clinical encounters; high quality counselling may inform the preference someone holds for contraceptive use, complicating the temporality of our analysis. As such, our analysis of contraceptive counselling quality and preferences should not be interpreted causally. We also are not able to examine how state policy environments shape access to contraceptive care; given our sampling strategy, the state in which respondents live and their racial and ethnic identity are highly correlated. While this study is strengthened by the inclusion of people not currently using contraception who may face the most barriers to accessing contraception, the inclusion criteria for this study specified that if a respondent had not used contraception in the past year, they needed to have wanted to use contraception, making it more likely they desired a method they were not able to obtain. Indeed, many people selected multiple contraceptive methods they preferred to use, suggesting any/some methods may be perceived as better than none. Finally, we cannot tease apart whether any of the current users preferred dual method use – wanting to continue their current method and use another method concurrently (e.g. condoms).

## Conclusion

There are a multitude of reasons for, and barriers related to non-use of preferred contraceptive methods among the AANHPI, Black or African American, Indigenous, and Latina/Latinx respondents in this study including concerns about side effects, financial/logistical challenges, and low-quality provider interactions. These findings underscore the critical importance of person-centred contraceptive counselling for those who seek contraception from a clinic-based provider, recognising that people’s preferences for method features and experiences with contraceptive methods are unique. As such, contraceptive care delivery should be organised around expanding access to such care through a range of models (e.g. clinic-based, pharmacy-based, over-the-counter etc.) and ensuring that people have access to the contraceptive counselling they want and comprehensive information on methods so that they can explore and decide to use a(another) method when needed and desired. There is critical need for strategies to promote sharing of accurate information about contraceptive methods; person-centred care which considers the complexities of individual characteristics, circumstances, and contraceptive preferences; and increased physical and financial access to the full range of methods. Ensuring that people are able to fulfil their contraceptive desires and preferences is essential to realising reproductive autonomy.

## Supplementary Material

Appendix
