# Supplementary material for: Preferred use of contraceptive methods and reasons for non-use: a cross-sectional survey of a sample of Black, Indigenous, and people of colour in the United States
Source: Sex Reprod Health Matters. 2025 Apr 16;33(1):2494418. doi: 10.1080/26410397.2025.2494418 (PMC12203685; doi:10.1080/26410397.2025.2494418)
Supplement: Appendix [file ZRHM_A_2494418_SM1341.docx]

Appendix

*Table A1; List of survey response options for reasons not using the birth control method preferred and categories to describe these reasons*

| **Reasons** |
| --- |
| **Method-related** |
| Worried about side effects and/or health risks |
| Need more information about methods |
| **Logistical/financial** |
| Too expensive and/or no insurance |
| Challenges with transportation |
| Challenge making an appointment |
| Don't have a regular doctor or clinic |
| **Sexual relationships** |
| Partner won't let me or doesn't want me to |
| Infrequent sex |
| **Situational/relational** |
| Privacy concerns |
| Fear being judged |
| Didn't feel comfortable/safe getting my method |
| **Provider-related** |
| My provider advised me against it |
| Treated unfairly by staff at the doctor's office |
| Couldn't use my preferred language when talking |
| **Trying to get pregnant/currently pregnant/just had baby** |
| **Other** |
| Covid-19-related reason |
| Other |
| Prefer not to answer |

*Table A2: Rating the most recent experience with a healthcare provider or pharmacist related to birth control care in the past 12 months (%) (n=438)*

| Interpersonal quality index (mean: 3.8, range: 1.25 - 5) (n=402) |  |  |  |  |
| --- | --- | --- | --- | --- |
|  | **Asked me what matters to me in my method** | **Gave me enough information to make best decision** | **They kept my information private** | **They answered all my questions** |
| *Rating* |  |  |  |  |
| Generally positive (excellent, very good, good) | 73% | 69% | 98% | 84% |
| Generally negative (fair, poor | 27% | 31% | 2% | 16% |

Table excludes participants who did not select an answer to each respective question.

*Figure A1: Distribution of interpersonal quality index scores*

**

*Table A3: Reasons for not using preferred method(s) among people not using preferred method, by method type preferred among current contraceptive users*

|  | **Preferred method type** | | | | | |
| --- | --- | --- | --- | --- | --- | --- |
| **Reasons** | **Overall**  **(N=174)** | **LARC**  **(N=97)** | **SARC**  **(N=56)** | **Permanent**  **(N=37)** | **Barrier**  **(N=29)** | **Natural**  **(N=17)** |
|  |  |  |  |  |  |  |
| **Method-related** | 61.5% | 79.4% | 67.9% | 56.8% | 37.9% | 29.4% |
| Worried about side effects and/or health risks | 59.8% | 78.4% | 66.1% | 56.8% | 37.9% | 23.5% |
| Need more information about methods | 2.9% | 2.1% | 1.8% | 5.4% | 0% | 5.9% |
| **Logistical/financial** | 40.2% | 43.3% | 46.4% | 59.5% | 20.7% | 11.8% |
| Too expensive and/or no insurance | 26.4% | 26.8% | 37.5% | 43.2% | 17.2% | 11.8% |
| Challenges with transportation | 6.3% | 10.3% | 8.9% | 2.7% | 0% | 0% |
| Challenge making an appointment | 13.2% | 15.5% | 14.3% | 16.2% | 3.4% | 0% |
| Don't have a regular doctor or clinic | 8.6% | 12.4% | 10.7% | 8.1% | 10.3% | 11.8% |
| **Sexual relationships** | 20.7% | 17.5% | 17.9% | 21.6% | 55.2% | 17.6% |
| Partner won't let me or doesn't want me to | 6.9% | 2.1% | 1.8% | 8.1% | 27.6% | 11.8% |
| Infrequent sex | 15.5% | 16.5% | 16.1% | 16.2% | 34.5% | 5.9% |
| **Situational/relational** | 25.3% | 29.9% | 30.4% | 24.3% | 20.7% | 23.5% |
| Privacy concerns | 8.6% | 8.2% | 14.3% | 5.4% | 13.8% | 5.9% |
| Fear being judged | 10.9% | 11.3% | 12.5% | 13.5% | 13.8% | 17.6% |
| Didn't feel comfortable/safe getting my method | 15.5% | 20.6% | 16.1% | 16.2% | 10.3% | 11.8% |
| **Provider-related** | 13.2% | 14.4% | 7.1% | 21.6% | 13.8% | 17.6% |
| My provider advised me against it | 12.1% | 13.4% | 7.1% | 21.6% | 10.3% | 17.6% |
| Treated unfairly by staff at the doctor's office | 1.1% | 1% | 0% | 0% | 3.4% | 0% |
| Couldn't use my preferred language when talking | 0% | 0% | 0% | 0% | 0% | 0% |
| **Trying to get pregnant/currently pregnant/just had baby** | 1.7% | 0% | 1.8% | 2.7% | 6.9% | 5.9% |
| **Other** |  |  |  |  |  |  |
| Covid-19-related reason | 3.4% | 4.1% | 1.8% | 5.4% | 0% | 0% |
| Other | 4.6% | 4.1% | 7.1% | 5.4% | 3.4% | 17.6% |
| Prefer not to answer | 2.3% | 2.1% | 3.6% | 2.7% | 0% | 5.9% |

Respondents could select all specific reasons that applied.

*Table A4: Reasons for not using preferred method(s) among people not using preferred method, by method type preferred among non-current contraceptive users*

|  | **Preferred method type** | | | | | |
| --- | --- | --- | --- | --- | --- | --- |
| **Reasons** | **Overall**  **(N=92)** | **LARC**  **(N=47)** | **SARC**  **(N=54)** | **Permanent**  **(N=13)** | **Barrier**  **(N=22)** | **Natural**  **(N=11)** |
| **Method-related** | 71.7% | 85.1% | 77.8% | 61.5% | 50% | 45.5% |
| Worried about side effects and/or health risks | 71.7% | 85.1% | 77.8% | 61.5% | 50% | 45.5% |
| Need more information about methods | 3.3% | 2.1% | 1.9% | 0% | 0% | 18.2% |
| **Logistical/financial** | 45.7% | 51.1% | 50.0% | 76.9% | 50% | 54.5% |
| Too expensive and/or no insurance | 22.8% | 21.3% | 20.4% | 46.2% | 31.8% | 54.5% |
| Challenges with transportation | 14.1% | 12.8% | 16.7% | 23.1% | 9.1% | 18.2% |
| Challenge making an appointment | 18.5% | 21.3% | 18.5% | 23.1% | 22.7% | 9.1% |
| Don't have a regular doctor or clinic | 16.3% | 19.1% | 20.4% | 15.4% | 13.6% | 18.2% |
| **Sexual relationships** | 52.2% | 48.9% | 50.0% | 76.9% | 68.2% | 72.7% |
| Partner won't let me or doesn't want me to | 4.3% | 0% | 7.4% | 7.7% | 9.1% | 0% |
| Infrequent sex | 51.1% | 48.9% | 48.1% | 76.9% | 68.2% | 72.7% |
| **Situational/relational** | 33.7% | 19.1% | 42.6% | 15.4% | 40.9% | 27.3% |
| Privacy concerns | 14.1% | 8.5% | 18.5% | 15.4% | 13.6% | 9.1% |
| Fear being judged | 20.7% | 8.5% | 27.8% | 7.7% | 27.3% | 27.3% |
| Didn't feel comfortable/safe getting my method | 15.2% | 12.8% | 16.7% | 0% | 18.2% | 0% |
| **Provider-related** | 9.8% | 10.6% | 13.0% | 15.4% | 13.6% | 9.1% |
| My provider advised me against it | 4.3% | 4.3% | 7.4% | 0% | 4.5% | 0% |
| Treated unfairly by staff at the doctor's office | 7.6% | 8.5% | 9.3% | 15.4% | 13.6% | 9.1% |
| Couldn't use my preferred language when talking | 1.1% | 0% | 1.9% | 0% | 0% | 0% |
| **Trying to get pregnant/currently pregnant/just had baby** | 2.2% | 4.3% | 0% | 7.7% | 0% | 9.1% |
| **Other** |  |  |  |  |  |  |
| Covid-19-related reason | 3.3% | 4.3% | 1.9% | 0% | 4.5% | 0% |
| Other | 1.1% | 0% | 0% | 0% | 0% | 9.1% |
| Prefer not to answer | 0% | 0% | 0% | 0% | 0% | 0% |

Respondents could select all specific reasons that applied.
